# Antibiotic Activity Potentiation and Physicochemical Characterization of the Fixed *Orbignya speciosa* Almond Oil against MDR *Staphylococcus aureus* and Other Bacteria

**DOI:** 10.3390/antibiotics8010028

**Published:** 2019-03-17

**Authors:** Jean Ferreira Machado, Maria do Socorro Costa, Saulo Relison Tintino, Fábio Fernandes Galvão Rodrigues, Camila Bezerra Nobre, Henrique Douglas Melo Coutinho, José Galberto Martins da Costa, Irwin Rose Alencar de Menezes, Erlânio Oliveira de Sousa

**Affiliations:** 1 Technology Center, Faculty of Technology Cariri, Juazeiro do Norte 63041-190, Brazil; jean.lavras@gmail.com (J.F.M.); erlaniourca@hotmail.com (E.O.d.S.); 2 Department of Biological Chemistry, Regional University of Cariri, Crato 63105-000, CE, Brazil; corrinha_live@yahoo.com.br (M.d.S.C.); saulorelison@gmail.com (S.R.T.); fabiogalvao01@hotmail.com (F.F.G.R.); galberto.martins@gmail.com (J.G.M.d.C.); irwinalencar@yahoo.com.br (I.R.A.d.M.); 3 Faculty of Medicine of Juazeiro do Norte, Juazeiro do Norte 63041-190, Brazil; milinhanobre@gmail.com

**Keywords:** *Orbignya speciosa*, babassu, fatty acid, anti-bacterial, aminoglycosides

## Abstract

*Orbignya speciosa* (babassu) is an important palm tree in Brazil whose fixed almond oil is used in popular medicine and especially in food, in addition to being a research target for the manufacture of biofuels. The aim of this study was to evaluate the fixed almond oil physicochemical characterization and its antibacterial activity in isolation and in association with aminoglycosides against standard and multidrug-resistant bacteria. Analyses such as water content, pH, acidity, peroxide index, relative density, and refractive index indicate the stability and chemical quality of the oil. In the oil’s GC/MS chemical composition analysis, a high saturated fatty acid (76.90%) content was observed. Lauric acid (56.28%) and oleic acid (23.10%) were the major oil components. In the antibacterial test, a more significant oil activity was observed against *K. pneumoniae* KP-ATCC 10031 (minimal inhibitory concentration (MIC) = 406.37 μg/mL) and *Staphylococcus aureus* ATCC 6538 (MIC = 812.75 μg/mL), but for the other strains—including standard and multi-resistant strains—the oil presented an MIC ≥ 1024 μg/mL. Furthermore, a synergistic effect was observed when the oil was associated with amikacin and gentamicin against *S. aureus* (SA-10) and an antagonistic effect was observed with amikacin against *Escherichia coli*. Data indicate the *O. speciosa* oil as a valuable nutritional source of lauric, oleic, and myristic fatty acids with an ability to modulate aminoglycoside activity.

## 1. Introduction

The growing increase in microorganismal resistance to conventional antimicrobial drugs such as aminoglycosides has been challenging the scientific community and causing serious public health risks [1]. Aminoglycosides are a class of antibiotics which have been used to treat many bacterial infections, and possess a mechanism of action based on protein synthesis inhibition by binding to the prokaryotic 30S ribosome, preventing adequate mRNA translation [2,3].

Aminoglycosides have a broad bactericidal spectrum, and despite their benefits in treating many infectious disorders, bacterial resistance to them has become a problem in recent decades [3]. One of the problems associated with aminoglycoside resistance is the serious toxic effects associated with high doses or chronic treatment, leading to ototoxicity and/or nephrotoxicity [4].

Due to the growing resistance against aminoglycosides, several natural plant products have been studied regarding their antibacterial and antibiotic modifying activities. These natural products can increase or decrease an antibiotic’s activity, thus being an interesting tool against the resistance problem [1,4].

*Orbignya speciosa* (babassu) is one of the most important palms in Brazil because its parts are used to provide food, fuel, fibers, and building materials, among other purposes. The babassu mesocarp flour is widely used for the treatment of chronic wounds, colitis, duodenal ulcers, arthritis, menstrual cramps, nervous exhaustion, cellulitis, varicose veins, and tumors [5].

Babassu presents itself as an important resource that is popularly used to produce fixed oils for centuries, being a prominent species for more than 300,000 extractive families who have their main source of income from the manual fruit peeling process to remove the almond [6]. The fixed oil represents 65% of the seed, and is used for the manufacture of soaps, surfactants, and margarines [7].

The fixed babassu almond oil is usually artisanally extracted, and the generated residues are a source of organic matter that serve as a source of proteins, fibers, minerals, and vitamins [6,7]. However, the mechanical oil extraction through almond pressing is the main method used by industries and corporations, this being less aggressive than the artisanal process, which minimizes oil oxidation providing a longer shelf life and conservation [8].

With this in mind, the objective of this study was to conduct a physicochemical characterization and evaluate the antibacterial and aminoglycoside antibiotic modifying activity of the *O. speciosa* fixed oil obtained by cold pressing.

## 2. Materials and Methods

### 2.1. Botanical Collection and Identification

*Orbignya speciosa* (babassu) fruits were collected from an area in the Chapada do Araripe (Sítio Arajara), Municipality of Barbalha, Ceará, Brazil. An exsiccate (#9709) of the species is found in the Caririense Dárdano de Andrade Lima Herbarium (HCDAL) of the Regional University of Cariri (URCA).

### 2.2. Acquiring the Fixed Oil

The fixed oil was obtained using the mechanical extraction method with discontinuous hydraulic pressing using 500 g of *O. speciosa* almonds. The sample was added to a stainless-steel cylinder and pressed for approximately 2 h, the pressure of which was recorded by a manometer at 15 T. The fixed oil obtained yielded 48.0% of the crude material, and was stored in a hermetically sealed amber flask and kept refrigerated.

### 2.3. Physicochemical Characterization and Fatty Acid Analysis

Physicochemical analysis was performed according to protocols described by the Instituto Adolfo Lutz [8].

Fatty acid analysis was performed as described by Pereira et al. [9]. The individual components were identified by matching their mass spectra with those of a database with the library constructed using the spectrometer (Wiley, 229) and NIST 08 using retention indices (IRs) as a pre-selection [10], as well as by visually comparing standard fragmentation to those reported in the literature [11].

### 2.4. Antibacterial Analysis

#### 2.4.1. Strains Utilized

Standard and multidrug-resistant bacterial strains were used in the analyses. The standard strains were: *Staphylococcus aureus* SA–ATCC 6538, *Bacillus cereus* BC–ATCC 33018, *Escherichia coli* EC–ATCC 10536, *Pseudomonas aeruginosa* PA–ATCC 9027, *Klebsiella pneumoniae* KP–ATCC 10031, *Shigella flexneri* EC–ATCC 12022, and *Proteus vulgaris* PV–ATCC 13315. The multiresistant strains were: *S. aureus* SA–10 and *E*. *coli* EC–06, with resistance profiles identified in Table 1. Bacteria were maintained in blood agar base (Laboratory Difco Ltd., Franklin Lakes, USA) and cultured at 37 °C for 24 h in heart infusion agar (HIA, Difco Laboratories Ltd., Franklin Lakes, USA).

#### 2.4.2. Drugs

The drugs used were the aminoglycosides amikacin and gentamicin (Sigma Co., St. Louis, MO, USA). The drugs were prepared at the concentration of 5000 μL/mL and diluted in sterile water.

#### 2.4.3. Minimum Inhibitory Concentration Test

The fixed oil minimal inhibitory concentration (MIC) was determined according to Pereira et al. [9]. For the assay, Eppendorf tubes were prepared using 100 μL of the inoculum and 900 μL of 10% BHI (Brain Heart Infusion) liquid culture medium in 96-well plates filled in the numerical sense with 100 μL of this solution being added to each well. Serial microdilution was then performed using 100 μL of the oil, varying the concentration from 1024 to 1.0 μg/mL. The tests were performed in triplicates, and the plates were incubated for 24 h at 37 °C. To read the bacterial MIC, 20 μL of resazurin was added to each well, where the color of the wells were observed after 1 h with a color change from blue to red corresponding to microbial growth and a permanence in the blue color corresponding to the absence of growth.

#### 2.4.4. Antibiotic Activity Modifying Effect

Evaluation of the fixed oil as an antibiotic activity modifier was performed according to Pereira et al. [9]. For each Eppendorf tube, 1162 μL of 10% BHI was used with 150 μL of the inoculum from each strain as well as the tested oil at a volume corresponding to a sub-inhibitory concentration (MIC/8 = 128 μg/mL). Controls were prepared with only 1350 μL of 10% BHI and 150 μL of bacterial suspension. The plates were filled in numerical order, and each well received 100 μL of this solution. Microdilution was performed with 100 μL of each antibiotic being added up to the penultimate well and the final volumes being discarded. The tests were performed in triplicates, and the plates were incubated at 37 °C for 24 h and read through the addition of resazurin.

### 2.5. Statistical Analysis

Statistical analysis of the microbiological assays was performed using the statistical program GraphPad Prism 5.0. The geometric means were analyzed by a two-way ANOVA followed by Bonferroni’s post-hoc test (where *p* < 0.05 was considered significant). All assays were performed in triplicate.

## 3. Results and Discussion

### 3.1. Physicochemical Oil Analysis and Fatty Acid Profile 

Results from the *O. speciosa* fixed almond oil physicochemical characterization are expressed in Table 2. The oil presented a moisture content of 0.90%, representing a minimum percentage of non-combined water (<1%), suggesting high oil quality and durability [12]. Water content values lower than 1.0% were observed for the babassu fixed oils in the municipalities of São Luís and Imperatriz do Maranhão, Northeast Brazil [13].

The 4.60% acid value reflects stability against neutralization and the 4.40 meq/kg peroxide index value indicates greater resistance to oxidation. The pH value of 6.41 is an important parameter, since it indicates the inhibitory range of several bacterial strains [12]. The refraction index was 1.46, with this criterion being widely used as a quality and oil identity criterion. Fixed babassu oil samples obtained artisanally from municipalities in the state of Maranhão, Northeast Brazil, had a refractive index in the range 1.4475–1.4501 [13]. The direct density measurement found was 0.30 (g/cm³).

The *O. speciosa* fixed oil constituents, their percentage composition, and their retention indices (RIs) are presented in Table 3. The oil’s GC/MS chemical composition analyses allowed the identification of four constituents, corresponding to 100% of the fatty acids present. The oil was characterized by a high saturated fatty acid content (76.90%), where lauric acid was the most representative (56.28%), followed by myristic acid (14.38%). The only unsaturated fatty acid identified was oleic acid (23.10%).

In collaboration with these results, *O. speciosa* oil has been reported to have a predominance of saturated fatty acids and intermediate carbon chains (C6 through to C16), of which 40%–55% of the composition is lauric acid [14,15]. Due to this, the oil exhibits significant characteristics for industrial use, as it is resistant to thermal oxidation and has a low melting temperature [16].

The major constituents observed in the fixed *O. speciosa* oil in this study were different from those found in the hot oil extracted using an organic solvent. Darnet et al. [17] found oleic (75.7%) and palmitic (18.9%) acids at higher quantities in the oil. Similarly, Silva et al. [6] found oleic (74.0%) and palmitic (16.7%) acids as the main constituents of this oil. In contrast, unsaturated fatty acids were predominant in the oil obtained in both studies.

### 3.2. Antibacterial Activity and Antibiotic Modifying Action

The antibacterial test results showed a more significant *O. speciosa* fixed oil activity against *K. pneumoniae* KP-ATCC 10031 (MIC = 406.37 μg/mL) and *S. aureus* SA-ATCC 6538 (MIC = 812.75 μg/mL), as shown in Table 4. For the other bacterial strains, including standard and multiresistant strains, the oil had an MIC ≥ 1024 μg/mL.

Several fixed oils have also been reported for their antibacterial potential. Costa et al. [18] showed the antibacterial activity of the fixed *Caryocar coriaceum* pulp (pequi) oil against several bacterial strains. Saraiva et al. [3] observed significant antibacterial activity of the pequi pulp fixed oil against *S. aureus* and *E. coli* multiresistant strains (MIC = 512 μg/mL). The *Malus sp.* (macieira) seed oil also presented activity against standard *E. coli* and *S. aureus* strains (MIC = 521 μg/mL) [19].

**Figure 1:**
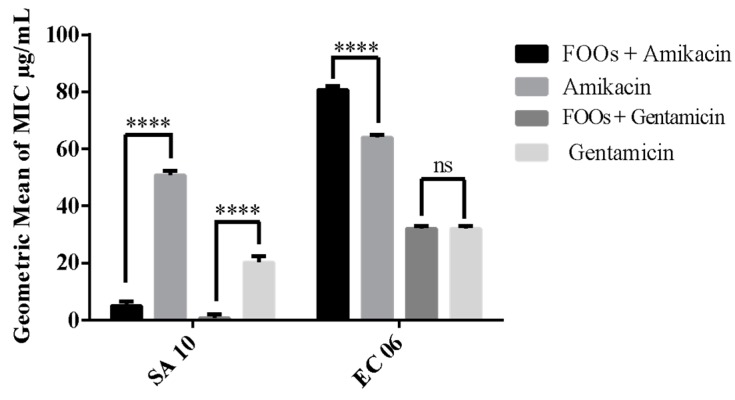
Effect of fixed oil of *Orbignya speciosa* (FOOs) on the activity of aminoglycoside antibiotics against multiresistant strains of *Staphylococcus aureus* (SA) and *Escherichia coli* (EC). Values represent the geometric mean ± M.S.E. (mean standard error). One-way ANOVA, followed by the Bonferroni test. **** statistically significant value with *p* < 0.0001 antibiotic + FOOs vs. control (antibiotic); ns: not statistically significant.

Figure 1 shows the MICs of the antibiotics and the synergistic effects of the fixed oil in association with aminoglycoside antibiotics. A significant synergistic MIC reduction effect was observed with amikacin and gentamicin against *S. aureus* (SA-10). On the other hand, a significant antagonistic effect was observed in the amikacin activity against *E. coli*. The oil’s modifying activity over aminoglycoside antibiotics varied according to the antibiotic type associated with the oil and the bacterial strain analyzed.

The fixed oil’s ability to act either as an antibacterial or as an antibiotic modifier may be related to the detergent property of fatty acids against the amphipathic structure of the bacterial cell membrane. In addition, the detergent ability to solubilize membrane components (lipids and proteins) may cause breaks in this structure and affect metabolic processes essential for obtaining energy for the bacterial cell, such as the electron transport chain and oxidative phosphorylation. These membrane damages can also lead to difficulties with nutrient absorption, enzymatic activity inhibition, and toxic peroxidation generation [3,20,21]. In addition, the presence of hydrophobic compounds in the fixed oils may make the cell more permeable to antibiotics, thus increasing its efficiency and reducing the minimum concentration required to affect the bacterial cell [3], also affecting bacterial efflux pumps [22]. 

Many reports have demonstrated the effect of fixed oils from animals and plants enhancing antibiotic activity, such as *Caryocar coriaceum*, *Rhinella jimi* and others [3,23]. The use of oils conferring a synergistic action (MIC reduction) of antibiotics is a viable alternative, in clinical terms, against bacterial resistance. 

The use of oils conferring a synergistic action (MIC reduction) of antibiotics is a viable alternative against bacterial resistance in several infectious disease cases [24]. The synergistic action of the fixed oils can occur in different ways, including a reduction in antibiotic accumulation within the bacterial cell, either by a decrease in outer membrane permeability or by drug efflux to the extracellular environment [24,25].

## 4. Conclusions

The data obtained indicate the fixed *O. speciosa* almond oil as a valuable source of lauric, oleic, and myristic fatty acids, with a potential for modifying aminoglycoside antibiotic activity. In this sense, the use of the almonds which become residues during babassu consumption or processing, as a source for obtaining the fixed oil with the purpose of studying its antibacterial properties in isolation and in association with antibiotics is suggested.

## Figures and Tables

**Table 1:** Bacterial source and antibiotic resistance profile.

Bacteria	Source	Resistance Profile
*S. aureus* (SA–10)	Surgical wound	Cephalothin, cephalexin, cefadroxil, oxacillin, penicillin, ampicillin, ampicillin + sulbactam, amoxicillin, moxifloxacin, ciprofloxacin, levofloxacin, erythromycin, clarithromycin azithromycin and clindamycin
*E. coli* (EC–06)	Surgical wound	Cephalothin, cephalexin, cefadroxil, ceftriaxone, cefepime and ampicillin + sulbactam

**Table 2:** Physicochemical properties of the fixed oils of the almond of *O. speciosa*.

Physicochemical Properties	Values
Water content (% p/p)	0.90 ± 0.60
pH	6.41 ± 1.00
Acidity (as oleic acid %)	4.60 ± 0.95
Relative density (g/cm³)	0.30 ± 0.45
Peroxide index (meq/Kg)	4.40 ± 0.90
Refraction index (40 °C)	1.46 ± 0.25

Results are expressed with means ± S.E.M. (*n* = 3) of experiments performed in triplicate.

**Table 3:** Fatty acids identified in the fixed oil of the almond of *O. speciosa*.

Order	Constituents	* RI (min)	%
	Saturated		76.90
1	Lauric acid (C12:0)	16.943	56.28
2	Myristic acid (C14:0)	21.653	14.38
3	Palmitic acid (C16:0)	27.706	6.24
	Unsaturated		23.10
4	Oleic acid (C18:1)	31.181	23.10
Total identified			100.00

* relative retention indices [11].

**Table 4:** Minimum inhibitory concentration values (μg/mL) of the fixed oil of the almond of *O. speciosa*.

Bacterial Strains	MIC (µg/mL)
*Proteus vulgaris* PV–ATCC 13315	≥1024
*Klebsiella pneumoniae* KP–ATCC 10031	406.37
*Shigella flexneri* EC–ATCC 12022	≥1024
*Pseudomonas aeruginosa* PA–ATCC 9027	≥1024
*Escherichia coli* EC–ATCC 10536	≥1024
*Escherichia coli* EC–06	≥1024
*Bacillus cereus* BC–ATCC 33018	≥1024
*Staphylococcus aureus* SA–ATCC 6538	812.75
*Staphylococcus aureus* SA–10	≥1024
